# Bore-Sight Calibration of Multiple Laser Range Finders for Kinematic 3D Laser Scanning Systems

**DOI:** 10.3390/s150510292

**Published:** 2015-05-04

**Authors:** Jaehoon Jung, Jeonghyun Kim, Sanghyun Yoon, Sangmin Kim, Hyoungsig Cho, Changjae Kim, Joon Heo

**Affiliations:** 1 School of Civil and Environmental Engineering, College of Engineering, Yonsei University, 50 Yonsei-ro, Seodaemun-gu, Seoul 120-749, Korea; E-Mails: lionheart_kr@yonsei.ac.kr (J.J.); jhkim2014@yonsei.ac.kr (J.K.); yoonssa@yonsei.ac.kr (S.Y.); netgo82@yonsei.ac.kr (S.K.); f15kdaum@yonsei.ac.kr (H.C.); 2 Department of Civil and Environmental Engineering, College of Engineering, Myongji University, 116 Myongji-ro, Cheoin-gu, Yongin, Gyeonggy-do 449-728, Korea; E-Mail: cjkim@mju.ac.kr

**Keywords:** SLAM, laser scanner, point cloud, calibration, adjustment

## Abstract

The Simultaneous Localization and Mapping (SLAM) technique has been used for autonomous navigation of mobile systems; now, its applications have been extended to 3D data acquisition of indoor environments. In order to reconstruct 3D scenes of indoor space, the kinematic 3D laser scanning system, developed herein, carries three laser range finders (LRFs): one is mounted horizontally for system-position correction and the other two are mounted vertically to collect 3D point-cloud data of the surrounding environment along the system’s trajectory. However, the kinematic laser scanning results can be impaired by errors resulting from sensor misalignment. In the present study, the bore-sight calibration of multiple LRF sensors was performed using a specially designed double-deck calibration facility, which is composed of two half-circle-shaped aluminum frames. Moreover, in order to automatically achieve point-to-point correspondences between a scan point and the target center, a V-shaped target was designed as well. The bore-sight calibration parameters were estimated by a constrained least squares method, which iteratively minimizes the weighted sum of squares of residuals while constraining some highly-correlated parameters. The calibration performance was analyzed by means of a correlation matrix. After calibration, the visual inspection of mapped data and residual calculation confirmed the effectiveness of the proposed calibration approach.

## 1. Introduction

Three-dimensional (3D) reconstruction of building structures is an important task as it allows for representing and documenting the current status of building components and maintenance work [1,2,3,4]. To facilitate 3D data acquisition of existing structures, laser scanners, which are fast, simple to use, and yet highly accurate, are widely employed [5,6]. However, the conventional static laser scanning has some limitations on its operability, particularly in indoor environments due to the presence of clutter and occlusions: in order to scan complex indoor spaces without loss of information, surveyors need to change the scanner location many times, which incurs extra work for registration of separate point-cloud data [7]. Alternatively, a mobile-based kinematic mapping system that uses the Simultaneous Localization and Mapping (SLAM) technique has been considered. Typically, SLAM has been employed for autonomous navigation of mobile systems; now, its applications have been extended to 3D data acquisition in indoor environments.

One of the common methods for acquisition of dense 3D data is the use of a 2D Laser Range Finder (LRF). A mobile system equipped with an LRF sensor scans the surrounding environments on the 2D plane and uses that information to localize its position. While it is moving, the system provides for its trajectory in a consistent way. 3D data can be obtained by scanning the vertical profiles in the direction perpendicular to its trajectory and registering all scans in the same coordinate system. The necessary hardware can be obtained by adding vertically mounted LRF sensors to a mobile platform [8,9]. In this case, misalignment of multiple sensors causes a systematic contradiction between them, which negatively impacts the utility of sensor fusion algorithms [10].

Calibration is the process of estimating the parameters that need to be applied to correct actual measurements to their true values [11]. These parameters together constitute a calibration model, which can be used to correct systematic instrumental errors [12]. Typically, the parameters of geometric sensors can be decomposed into intrinsic and extrinsic parameters. The intrinsic parameters control how the sensor functions and samples the scene. The extrinsic parameters determine the position and orientation of the sensor relative to a reference coordinate system [13]. According to the parameter types, calibration also can be divided into intrinsic and extrinsic forms. Intrinsic calibration refers to the process of setting the magnitude of the output of a sensor to the magnitude of the input property within a specified accuracy and precision. Extrinsic calibration refers to the process of finding the location of the sensor with respect to some other reference frame. This is typically required in multi-sensor fusion, where the data from different sensors has to be registered in a single reference frame [10,14]. Thus far, extrinsic calibration of multiple sensors in mobile system has been studied mainly for determination of the relative transformation between an LRF and a rigidly-attached camera on a mobile system [15,16,17,18]. However, there have been very few works on calibration of multiple LRF sensors [19]. Underwood *et al.* [10] proposed a multi-LRF calibration framework that minimizes the sensor misalignment, but the process was for an outdoor mobile mapping system with known motion information using high-performance, but also high-cost Inertial Measurement Unit (IMU) and Global Positioning System (GPS)-based navigation: The 3D geometric primitive (a single vertical pole) was detected while the vehicle drove around the test site and was used for the calibration input. For a calibration without motion information, Antone and Friedman [20] presented an automated procedure that uses a specially-designed pyramid target for calibration of the 3D location and orientation of LRF position with respect to a fixed 3D reference frame. The method, however, was designed only for a single LRF calibration. The multi-LRF calibration, proposed by Choi *et al.* [19], estimates a relative pose of 2D LRF sensors using scan data on two orthogonal planes, but requires further verification of coinciding or perpendicular scan-line inputs for calibration.

In this research, a kinematic laser scanning system is introduced for 3D indoor point-cloud data acquisition. A new calibration facility and a mathematical model are also presented, and the calibration for all LRF sensors involved is conducted and evaluated. This research assumes that the intrinsic sensor calibration is completed, and focuses on the extrinsic calibration of multiple LRF sensors to identify the rigid transformation from each sensor frame to the platform body frame. The calibration process entails the following: (1) a mathematical adjustment model is developed to estimate the influencing systematic factors; (2) a double-deck calibration facility, which is specifically designed for multiple LRF sensors, is introduced; (3) the calibration facility is scanned by the developed scanning system, and a total station with high accuracy is used for the coordinate measurements; (4) the calibrated parameters are estimated using a constrained least squares method; and finally (5) these parameters are analyzed and evaluated by means of a correlation matrix of parameters, residual calculations, and visual inspection of the produced point cloud data.

## 2. System Description and Mathematical Model

### 2.1. Kinematic 3D Laser Scanning System

**Figure 1:**
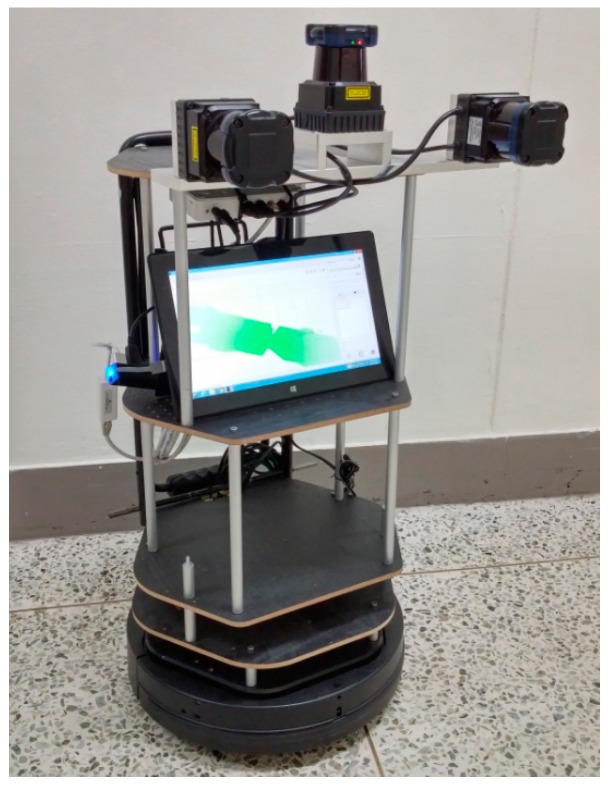
Kinematic 3D laser scanning system developed in this research.

Figure 1 shows the kinematic 3D laser scanning system developed in this research. It is approximately 35 cm (length) × 35 cm (width) × 78 cm (height). The platform carries three 2D LRFs (UTM-30LX, Hokuyo, Osaka, Japan). It provides a scanning range of 270° with angular resolution of 0.25°. It is able to measure distances up to 60 m, but without guaranteed reliability. One full scan cycle lasts for 25 ms, which supplies a 40 Hz measurement frequency. Data transfer to the host is realized through USB 2.0 interface or Ethernet cable. The measurement accuracy, according to the manufacturer [21], is defined as ±30 mm at 0.1 to 10 m range, and ±50 mm at 10 to 30 m range. Precision of the repeated measurement (standard deviation) is less than 10 mm at 0.1 to 10 m range, and less than 30 mm at 10 to 30 m range on white sheet [22]. In this research, one is mounted horizontally to update the system’s location, and the other two are mounted vertically to reconstruct the 3D scenes. In addition, a laptop computer is used for storing the data of each sensor, and an Ethernet hub used for connecting the three LRFs with the laptop computer.

### 2.2. Coordinate System

In almost all mapping scenarios, the sensors can sample only from a small region of the larger area to be mapped. The complete map is built by physically moving the sensors through the environments and registering the information into a single coordinate frame; this usually necessitates the transformation of several coordinate frames regardless of the sensors or mapping algorithm [10]. In the present study, three coordinate frames—the sensor, body, and global frames—were adopted to model the locations of the point acquisitions with respect to the location of the kinematic scanning system:

1. The sensor frame (*s*) of the 2D LRF provides point information by means of the distance and angle $p={\left[\begin{array}{ccc} \phi & \rho & 0 \end{array}\right]}^{\text{T}}$ in the sensor frame (*s*), which is then transformed to Cartesian coordinates as $p_{s}={\left[\begin{array}{ccc} \rho \cos(\phi ) & \rho sin(\phi ) & 0 \end{array}\right]}^{\text{T}}$. The sensor frame is defined by its alignment on the platform body frame.
2. The body frame (*b*) of the developed system is right-handed. Its origin is fixed to the center point on the mobile system, and the *x*-axis points in the direction of the platform’s forward movement. Each sensor is located with respect to the body frame by the lever-arm (three constant translation) and bore-sight (three rotation) parameters given by ${\text{T}}_{b}={\left[\begin{array}{ccc} x_{b} & y_{b} & z_{b} \end{array}\right]}^{\text{T}}$ and ${\text{R}}_{b}={\left[\begin{array}{ccc} \omega_{b} & \varphi_{b} & \kappa_{b} \end{array}\right]}^{\text{T}}$. In the present study, three different lever-arm and bore-sight parameter groups are used to define the middle-horizontal, left-vertical, and right-vertical LRF sensors.
3. The global frame (*g*) is fixed to an arbitrary point on the Earth and is used to represent the stationary environment in which the platform moves. In the present study, the origin of the global frame was fixed to the point from which the platform starts to move. The movement of the system with respect to the global frame is given by the three constant translation parameters ${\left[\begin{array}{ccc} x_{g} & y_{g} & z_{g} \end{array}\right]}^{\text{T}}$ and three rotation parameters ${\left[\begin{array}{ccc} \omega_{g} & \varphi_{g} & \kappa_{g} \end{array}\right]}^{\text{T}}$, respectively. In its practical implementation, however, the movement of the system (X) is limited to the 2D space and thus represented by two translation parameters on the *x*-*y* plane and one rotation parameter along the *z*-axis: $\text{X}={\left[\begin{array}{ccc} x_{g} & y_{g} & \kappa_{g} \end{array}\right]}^{\text{T}}$.

**Figure 2:**
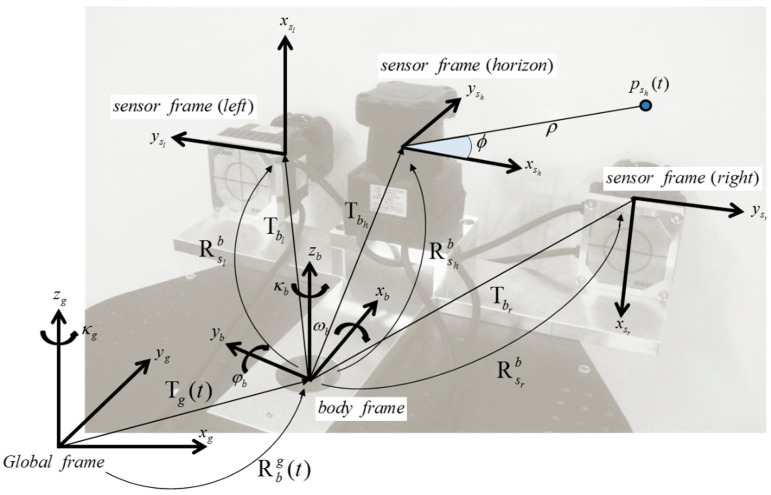
Configurations of sensor, body, and global frames of the developed system.

Figure 2 shows the configurations of the three coordinate systems. The three 2D LRFs used in the developed kinematic scanning system are mounted to point in three different directions. The middle LRF is mounted horizontally to sweep the *x-y* plane of the body frame and to correct the system’s position. The other two LRFs are mounted vertically (on the *y-z* plane of the body frame) to scan the profiles of the surrounding environments. 3D point clouds are obtained by registering those vertical profiles on the system’s trajectory in the global frame. Since the maximum field of view of the Hokuyo UTM-30LX is limited to 270°, two sensors are needed to capture the complete scan (360°) without a servo motor.

By combining two coordinate transformations, a point $p_{s_{i}}={\left[\begin{array}{ccc} x & y & 0 \end{array}\right]}^{\text{T}}$, in which *i* refers to the middle-horizontal (*h*), left-vertical (*l*), and right-vertical (*r*) scanners respectively on the 2D sensor frame, can be transformed to a point $p_{g}={\left[\begin{array}{ccc} x & y & z \end{array}\right]}^{\text{T}}$ on the 3D global frame. At time *t*, the transformation model is given by:

(1) $$p_{g}(t)=R_{b}^{g}(t)\left[{\text{R}}_{s_{i}}^{b}p_{s_{i}}(t)+{\text{T}}_{b_{i}}\right]+{\text{T}}_{g}(t)$$

where ${\text{R}}_{s_{i}}^{b}={\text{R}}_{\kappa_{i}}{\text{R}}_{\varphi_{i}}{\text{R}}_{\omega_{i}}$ is given by:

(2) $${\text{R}}_{\kappa_{i}}=\left[\begin{array}{ccc} \cos\kappa_{i} & -\sin\kappa_{i} & 0\\ \sin\kappa_{i} & \cos\kappa_{i} & 0\\ 0 & 0 & 1 \end{array}\right],\;{\text{R}}_{\varphi_{i}}=\left[\begin{array}{ccc} \cos\varphi_{i} & 0 & \sin\varphi_{i}\\ 0 & 1 & 0\\ -\sin\varphi_{i} & 0 & \cos\varphi_{i} \end{array}\right],\;{\text{R}}_{\omega_{i}}=\left[\begin{array}{ccc} 1 & 0 & 0\\ 0 & \cos\omega_{i} & -\sin\omega_{i}\\ 0 & \sin\omega_{i} & \cos\omega_{i} \end{array}\right]$$

and ${\text{T}}_{b_{i}}={\left[\begin{array}{ccc} x_{b_{i}} & y_{b_{i}} & z_{b_{i}} \end{array}\right]}^{\text{T}}$; both are determined from the *i*th mounted sensor position in the body frame, whereas ${\text{R}}_{b}^{g}(t)=\left[\begin{array}{ccc} \cos\kappa (t) & -\sin\kappa (t) & 0\\ \sin\kappa (t) & \cos\kappa (t) & 0\\ 0 & 0 & 1 \end{array}\right]$ and ${\text{T}}_{g}(t)={\left[\begin{array}{ccc} x(t) & y(t) & 0 \end{array}\right]}^{\text{T}}$ are determined by SLAM solution. 

This is due to the fact that the movement of the developed system is limited to the *x-y* plane, thus only two translation parameters (*x*(*t*), *y*(*t*)) and one rotation parameter (*κ*(*t*)) are considered. These equations are required whenever sensory information from a mobile system is mapped onto the global frame.

**Figure 3:**
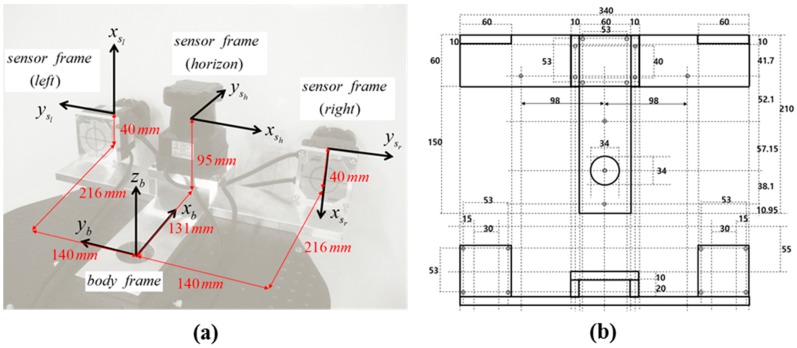
(**a**) Lever-arm parameters and (**b**) design drawing of the sensor stand.

For multi-sensor systems in SLAM, one of the primary sources of mapping error is sensor misalignment. Therefore, sensors must be carefully calibrated with respect to the body frame to enable the mobile system to accurately localize its position and map surrounding environments [20]. In Figure 2, the alignment errors in the body frame can be decomposed into lever-arm (*x_b_*, *y_b_*, *z_b_*) and bore-sight (*ω_b_*, *φ_b_*, *κ_b_*) errors. Compared with the lever-arm errors, whose impact is constant regardless to the range, the bore-sight errors are more substantial because they are accumulated with increasing range [23]. For example, bore-sight errors of only 1° can result in measurement errors of over 0.25 m at a distance of 15 m [24]. In most system installations, the lever-arms can be determined separately by physical means such as surveying instrument or design drawing [25,26,27], but the bore-sight can only be calculated indirectly (estimated by least squares calibration) [28]. Moreover, high correlation between the lever-arm and bore-sight parameters can lead to failure in the least squares calibration [27]; accordingly, the adjustment model in the present research assumes that the lever-arm parameters are known from the design drawing of the sensor stand (Figure 3), and therefore focuses only on the bore-sight calibration.

### 2.3. Calibration Facility

**Figure 4:**
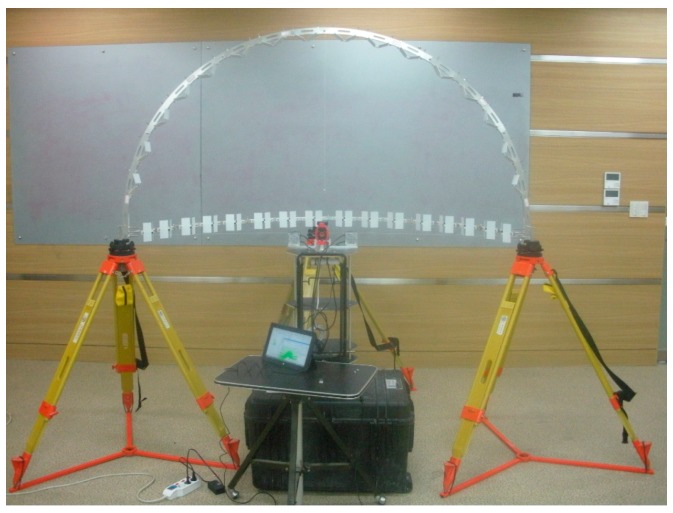
Establishment of the double-deck calibration facility.

**Figure 5:**
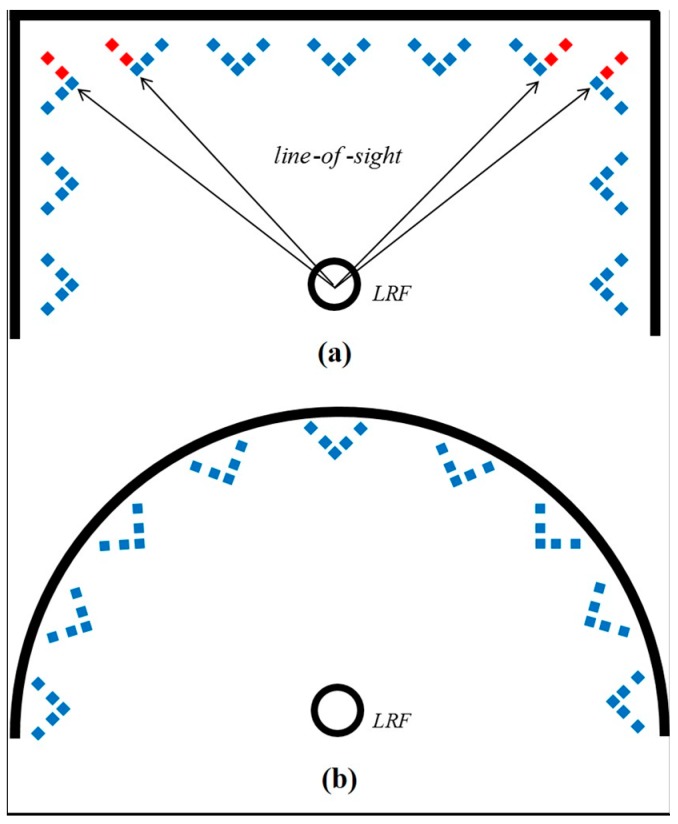
(**a**) Some targets are partially occluded (red dots) in the line-of-sight of the LRF sensor; and (**b**) no occlusions are detected using the half-circle frame.

For carrying out the bore-sight calibration, the 3D calibration facility was established as shown in Figure 4. In practice, the LRF’s large beam uncertainty at a long range creates obstacle to calibrate the LRF sensors in a large test site. Moreover, due to the LRF’s finite angular resolution, the calibration target should be placed within a small enough radius, thus producing a sufficient number of samples on the target surface for reliable estimation [20]. The multi-LRF calibration in a small area is also proved by Choi *et al.* [19]; the size of the calibration area is approximately 2 m × 2 m. In the present study, the calibration facility is composed of 1 m-radius aluminum frames hanging 16 targets on each (total: 32 targets). In addition, the half-circle shape was specifically designed, as shown in Figure 5, to avoid partial occlusions on the targets and to allow for a good network geometry of the target array. The latter is especially important, because weak network geometry of targets leads to correlation between calibration parameters in the adjustment [12,27]. The horizontal frame is supported by three tripods, and the vertical frame is wedged up using two screws on each side.

### 2.4. System Equations

**Figure 6:**
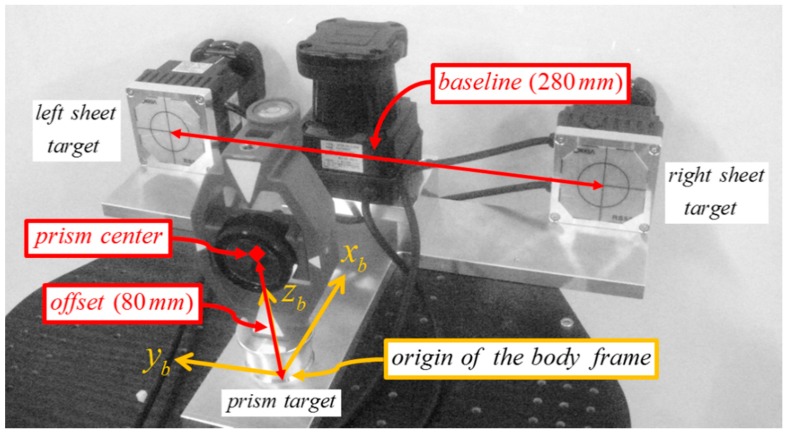
Prism and sheet targets for total station measurement.

Bore-sight errors usually are computed by least squares using a set of target points captured in the scanning areas [23]. In order to represent the full observation models of nonlinear transformations between the sensor and the global frame, Equation (1) is expanded as:

(3) $$\begin{array}{l} F_{x}=x_{g}+\cos\kappa_{g}(\cos\varphi_{b}\cos\kappa_{b}x_{s}-\cos\varphi_{b}\sin\kappa_{b}y_{s}+x_{b}+v_{off})\\ -\sin\kappa_{g}(\cos\varphi_{b}\sin\kappa_{b}x_{s}+\sin\omega_{b}\sin\varphi_{b}\cos\kappa_{b}x_{s}+\cos\omega_{b}\cos\kappa_{b}y_{s}-\sin\omega_{b}\sin\varphi_{b}\sin\kappa_{b}y_{s}+y_{b})\\ F_{y}=y_{g}+\sin\kappa_{g}(\cos\varphi_{b}\cos\kappa_{b}x_{s}-\cos\varphi_{b}\sin\kappa_{b}y_{s}+x_{b}+v_{off})\\ +\cos\kappa_{g}(\cos\varphi_{b}\sin\kappa_{b}x_{s}+\sin\omega_{b}\sin\varphi_{b}\cos\kappa_{b}x_{s}+\cos\omega_{b}\cos\kappa_{b}y_{s}-\sin\omega_{b}\sin\varphi_{b}\sin\kappa_{b}y_{s}+y_{b})\\ F_{z}=z_{g}+\sin\varphi_{b}\sin\kappa_{b}x_{s}-\cos\omega_{b}\sin\varphi_{b}\cos\kappa_{b}x_{s}+\sin\omega_{b}\cos\kappa_{b}y_{s}+\cos\omega_{b}\sin\varphi_{b}\sin\kappa_{b}y_{s}+z_{b} \end{array}$$

where ${\left[\begin{array}{cc} x_{s} & y_{s} \end{array}\right]}^{\text{T}}$ indicates a scanned point measured by an LRF in the sensor frame. Coordinate transformation from the sensor to the body frame is defined by the lever-arm ${\left[\begin{array}{ccc} x_{b} & y_{b} & z_{b} \end{array}\right]}^{\text{T}}$ and bore-sight ${\left[\begin{array}{ccc} \omega_{b} & \varphi_{b} & \kappa_{b} \end{array}\right]}^{\text{T}}$ parameters. In order to determine the system’s position from sensor frame to global frame ${\left[\begin{array}{ccc} x_{g} & y_{g} & z_{g} \end{array}\right]}^{\text{T}}$ and orientation $\kappa_{g}$ in the global frame, a prism target (set up on the origin of the body frame) and two specific reflective sheet targets (attached behind the vertical scanners’ stand) were used and measured by a total station. As shown in Figure 6, the system’s position was determined by subtraction of the offset (0.080 m) from the *z* value of the prism’s coordinates, and the system’s orientation was derived from the azimuth of the baseline connecting the two sheet targets’ centers.

**Figure 7:**
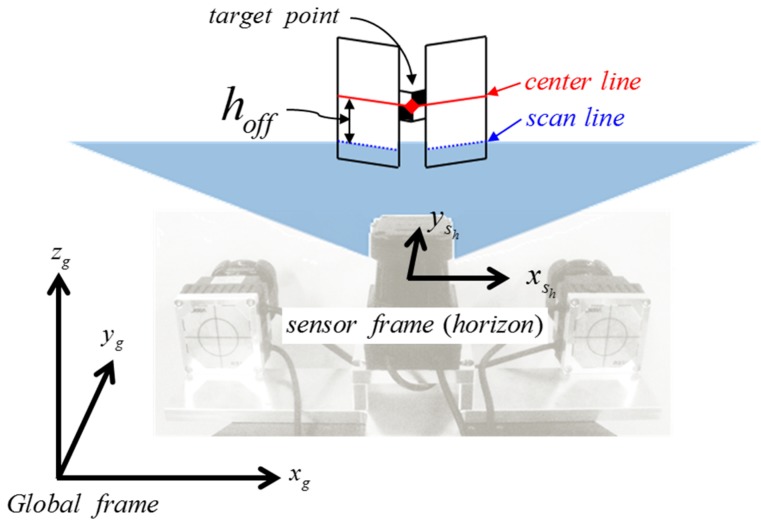
Conceptual figure of the horizontal target offset.

Additionally, two offset parameters, *h_off_* for horizontal scans and *v_off_* for vertical scans, should be determined prior to the adjustment. Calibration presumes the availability of targets with known coordinates determined with high precision [12]. *h_off_* and *v_off_* indicate the offsets between the transformed 2D scan lines and the target point in the 3D global frame. Figure 7 shows an example of horizontal scanning, in which *h_off_* represents the offset between the scan line and the center line: The former consists of the point returns from the target surface, and the latter passes the target point, dividing the target into halves. Likewise, *v_off_* is determined for two vertical scanners. Note that *v_off_* is assumed as same value for two vertical scanners after the system is properly leveled and aligned with the calibration facility. The alignment process and calibration facility will be explained in more detail in Section 2.3.

After the lever-arm $\left(x_{b},\;y_{b},\;z_{b}\right)$ and two offset $\left(h_{off},\;v_{off}\right)$ parameters are determined, only the bore-sight parameters $\left(\omega_{b},\;\varphi_{b},\;\kappa_{b}\right)$ remain to be estimated. The final adjustment model, which is designed to determine the nine bore-sight parameters for three different LRF sensors, is formed as:

(4) $$\left[\begin{array}{ccccccccc} \frac{\partial F_{x_{h}}}{\partial \omega_{h}} & \frac{\partial F_{x_{h}}}{\partial \varphi_{h}} & \frac{\partial F_{x_{h}}}{\partial \kappa_{h}} & & & & & & \\ \frac{\partial F_{y_{h}}}{\partial \omega_{h}} & \frac{\partial F_{y_{h}}}{\partial \varphi_{h}} & \frac{\partial F_{y_{h}}}{\partial \kappa_{h}} & & & & & & \\ \frac{\partial F_{z_{h}}}{\partial \omega_{h}} & \frac{\partial F_{z_{h}}}{\partial \varphi_{h}} & \frac{\partial F_{z_{h}}}{\partial \kappa_{h}} & & & & & & \\ & & & \frac{\partial F_{x_{l}}}{\partial \omega_{l}} & \frac{\partial F_{x_{l}}}{\partial \varphi_{l}} & \frac{\partial F_{x_{l}}}{\partial \kappa_{l}} & & & \\ & & & \frac{\partial F_{y_{l}}}{\partial \omega_{l}} & \frac{\partial F_{y_{l}}}{\partial \varphi_{l}} & \frac{\partial F_{y_{l}}}{\partial \kappa_{l}} & & & \\ & & & \frac{\partial F_{z_{l}}}{\partial \omega_{l}} & \frac{\partial F_{z_{l}}}{\partial \varphi_{l}} & \frac{\partial F_{z_{l}}}{\partial \kappa_{l}} & & & \\ & & & & & & \frac{\partial F_{x_{r}}}{\partial \omega_{r}} & \frac{\partial F_{x_{r}}}{\partial \varphi_{r}} & \frac{\partial F_{x_{r}}}{\partial \kappa_{r}}\\ & & & & & & \frac{\partial F_{y_{r}}}{\partial \omega_{r}} & \frac{\partial F_{y_{r}}}{\partial \varphi_{r}} & \frac{\partial F_{y_{r}}}{\partial \kappa_{r}}\\ & & & & & & \frac{\partial F_{z_{r}}}{\partial \omega_{r}} & \frac{\partial F_{z_{r}}}{\partial \varphi_{r}} & \frac{\partial F_{z_{r}}}{\partial \kappa_{r}} \end{array}\right]\left[\begin{array}{c} {\hat{\delta }}_{\omega_{h}}\\ {\hat{\delta }}_{\varphi_{h}}\\ {\hat{\delta }}_{\kappa_{h}}\\ {\hat{\delta }}_{\omega_{l}}\\ {\hat{\delta }}_{\varphi_{l}}\\ {\hat{\delta }}_{\kappa_{l}}\\ {\hat{\delta }}_{\omega_{r}}\\ {\hat{\delta }}_{\varphi_{r}}\\ {\hat{\delta }}_{\kappa_{r}} \end{array}\right]+\left[\begin{array}{c} u_{x_{h}}\\ u_{y_{h}}\\ u_{z_{h}}\\ u_{x_{l}}\\ u_{y_{l}}\\ u_{z_{l}}\\ u_{x_{r}}\\ u_{y_{r}}\\ u_{z_{r}} \end{array}\right]=\left[\begin{array}{c} {\hat{\upsilon }}_{x_{h}}\\ {\hat{\upsilon }}_{y_{h}}\\ {\hat{\upsilon }}_{z_{h}}\\ {\hat{\upsilon }}_{x_{l}}\\ {\hat{\upsilon }}_{y_{l}}\\ {\hat{\upsilon }}_{z_{l}}\\ {\hat{\upsilon }}_{x_{r}}\\ {\hat{\upsilon }}_{y_{r}}\\ {\hat{\upsilon }}_{z_{r}} \end{array}\right]$$

where the Jacobian matrix consists of partial derivatives of an observation group taken with respect to the *i*th sensor’s bore-sight parameter set ${\left[\begin{array}{ccc} \omega_{b} & \varphi_{b} & \kappa_{b} \end{array}\right]}_{i}^{\text{T}}$, $\hat{\delta }$ represents the vector of corrections to the approximate values of the bore-sight parameter set, *u* represents the mis-closure vector (the calculated minus the observed values), and $\hat{\upsilon }$ represents the estimated residuals [29].

The correct use of adjustment requires that some steps be taken to preclude the estimated parameters from being highly correlated and thus indeterminable [12]. For this reason, the constrained least squares method is employed to fix some correlated parameters in the adjustment. To formulate the matrix expression, the normal matrix and its matching constraints matrix are formed. In this procedure, the constraint equation borders the normal Equation (4) as:

(5) $$\left[\begin{array}{cc} J^{\text{T}}WJ & Z^{\text{T}}\\ Z & 0 \end{array}\right]\left[\begin{array}{c} \hat{\xi }\\ \hat{\lambda } \end{array}\right]=\left[\begin{array}{c} J^{\text{T}}W\tau \\ \tau_{c} \end{array}\right]$$

where *J* is the Jacobian matrix contains all of the coefficients of the linearized observations in Equation (4), *τ* is the observed minus computed values, and *W* is the weight matrix. Following this, observation equations for the constraints are included in the normal matrix as additional rows *Z* and columns *Z*^T^, and their constants 0 are added to the constants matrix as additional rows *τc*.$\hat{\xi }$ is the correction for the calibration parameters while *λ* is a Lagrangian multiplier [30]. The process is repeated until the corrections become sufficiently small.

## 3. Calibration Process

### 3.1. Calibration Facility Setup

**Figure 8:**
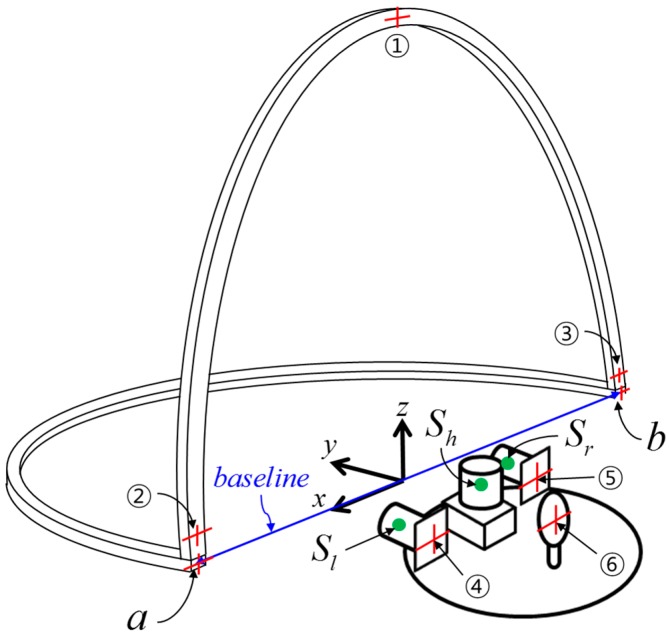
Temporary coordinate system for alignment.

**Figure 9:**
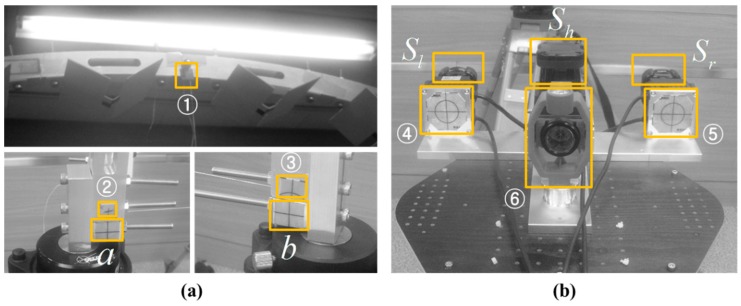
Total station measurement targets and LRF sensors (**a**) on the aluminum frames and (**b**) on the platform base.

After the assembly of the calibration facility, the aluminum frames would still be tilted and not aligned with the scanning system. For accurate calibration, therefore, its horizontal and vertical alignments with the scanning system should be ensured. In the experiment, the alignment was conducted using the temporary coordinate system shown in Figure 8 and total station measurement targets in Figure 9. 

Initially, the baseline connecting the two control points *a* and *b* of the horizontal frame was chosen to constitute the *x*-axis of the temporary coordinate system (hence their *y* and *z* values are 0 m), and the origin was centered on the baseline. The *y*-axis was orthogonal to the *x*-axis, pointing forward, and the *z*-axis was right-perpendicular to the *x-y* plane. Points ①, ②, ③ on the vertical aluminum frame (Figure 9a) were used to vertically adjust the orientation to correspond with the *x-z* plane. Points ④, ⑤, indicating the two reflective sheet targets attached behind the vertical scanners’ stand (Figure 9b), were used for alignment of the scanning system with respect to the vertical aluminum frame. Point ⑥, the prism target on the platform base (Figure 9b), was used to determine the origin of the body frame with respect to the temporary coordinate system. The scanner positions ($S_{h},\;S_{l},\;S_{r}$ ) with respect to the temporary coordinate system were used to determine the two offset values $h_{off}$ and $v_{off}$.

After the temporary coordinate system was defined, the scanning system and the horizontal aluminum frame were levelled by using a bull’s eye, and the vertical aluminum frame was vertically aligned by checking if the *y* coordinates of points ①, ②, ③ are the same, which allow the scan lines to be orthogonal to the targets’ surface. Then, the alignment of the scanning system with respect to the vertical aluminum frame was conducted by checking if the *y* coordinates of points ④, ⑤ are the same, which allows the vertical offset $v_{off}$ to be constant for every vertical target. All of the points were measured by a total station. The alignment results are listed in Table 1. Since the temporary coordinate system was defined according to the two control points *a* and *b*, the coordinates were fixed. All of the *y* values of points ①, ②, ③ were consistent (−0.001 m), indicating that the vertical mis-alignment error with the *x-z* plane was less than 0.001 m. Likewise, the same *y* values of points ④, ⑤ (−0.015 m) indicated that the mis-alignment error with the vertical aluminum frame also was less than 0.001 m.

After the alignment, the two offset values $h_{off}$ and $v_{off}$ were determined. The origin of the body frame was calculated by subtraction of the prism offset value (0.080 m) from the total station measurement of the prism target center (⑥). Then, the locations of each scanner were derived, as indicated in Table 1, from the design drawing of the sensor stand (Figure 3). In the temporary coordinate system, because the horizontal offset $h_{off}$ lay along the *z*-axis, it was equal to the difference between the *z* value of $S_{h}$ (0.045 m) and that of the horizontal targets (0 m), whereas the vertical offset $v_{off}$, which lay along the *y*-axis, was derived by subtraction of half the thickness of the vertical aluminum frame (0.010 m) from the *y* values of $S_{l}$ and $S_{r}$ (both were 0.050 m). Accordingly, $h_{off}$ and $v_{off}$ were determined to be 0.045 m and 0.040 m, respectively. Since the present study assumes that the lever-arm parameters are known, finally nine bore-sight parameters (three per each LRF sensor) remain for the adjustment process.

**Table 1:** Alignment results with respect to the temporary coordinate system (unit: M).

Point	*x*	*y*	*z*
a	1.030 (fixed)	0.000 (fixed)	0.000 (fixed)
b	−1.030 (fixed)	0.000 (fixed)	0.000 (fixed)
①	-	−0.001	-
②	-	−0.001	-
③	-	−0.001	-
④	0.139	−0.015	−0.009
⑤	−1.141	−0.015	−0.008
⑥	−0.001	−0.149	0.030
$S_{h}$	−0.001	−0.018	0.045
$S_{l}$	0.139	0.050	−0.009
$S_{r}$	−0.141	0.050	−0.008
**Offset**	**Value**		
$h_{off}$	0.045		
$v_{off}$	0.040		

**Figure 10:**
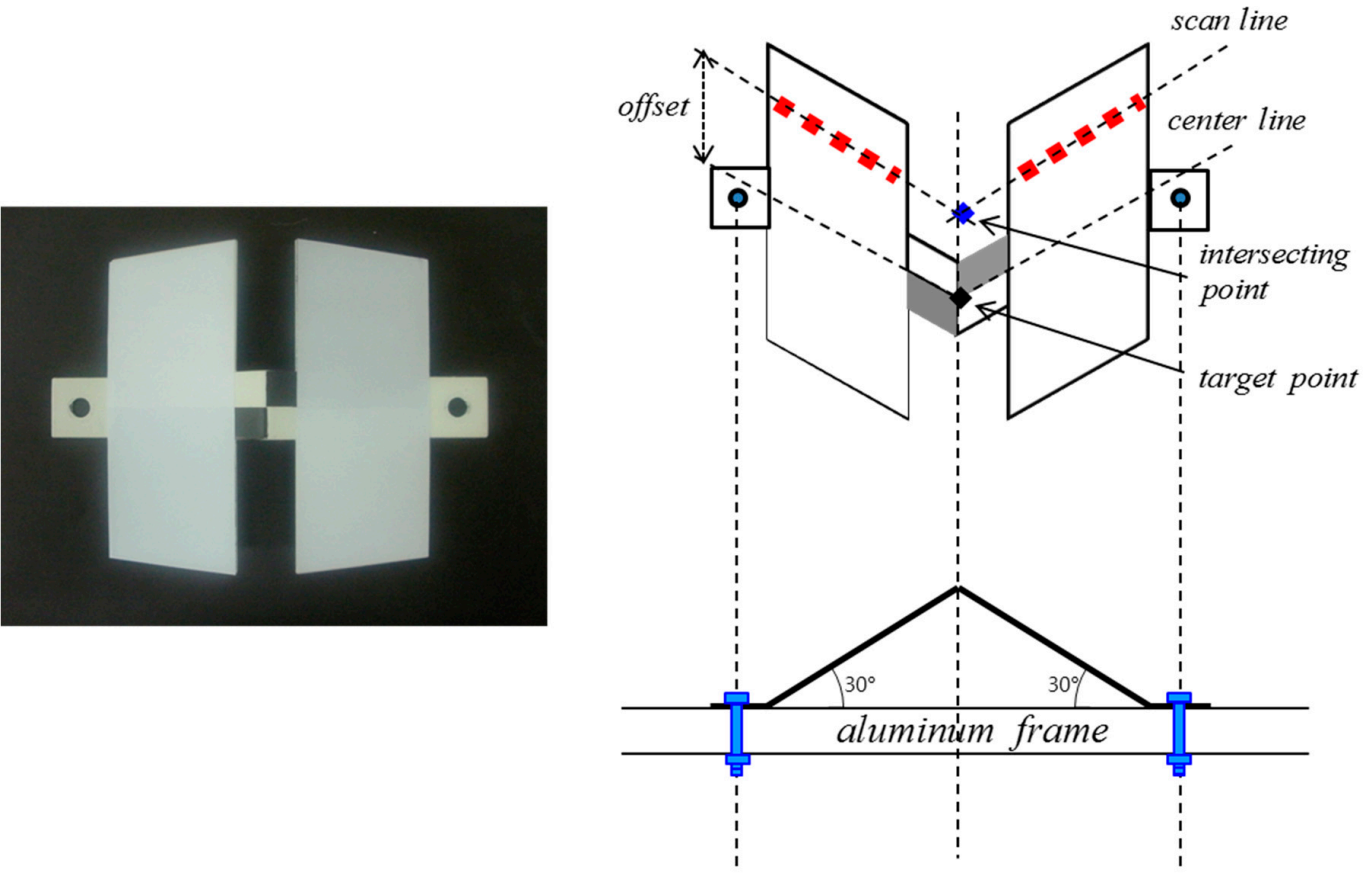
Target design for automated detection of target center.

Having redundancy in the adjustment model is crucial for quality assurance purposes when calibrating the scanner. However, since the scanned points are irregularly positioned on the target, it is rather difficult to achieve point-to-point correspondences between a scan point and the target center [31]. Alternatively, in order to automatically identify the target center from a scan line, a new aluminum target was specially designed, as shown in Figure 10. 

The target is composed of two panels adjoined at an angle of 30°. The size of each panel was chosen to ensure that at least seven scanned points are available within a distance of one meter. The joint part between two panels was hollowed so as to have no laser beam returned from this area: This allows acquirement of a separate scan line on each panel as well as the estimation of the coordinates of the intersecting point. By calculating the offset, the intersecting point can be matched to the target center. In scanning, all targets are kept stationary in the scanner’s field of view for a few minutes while a series of several hundred scans are obtained.

### 3.2. Determination of Target Coordinates

**Figure 11:**
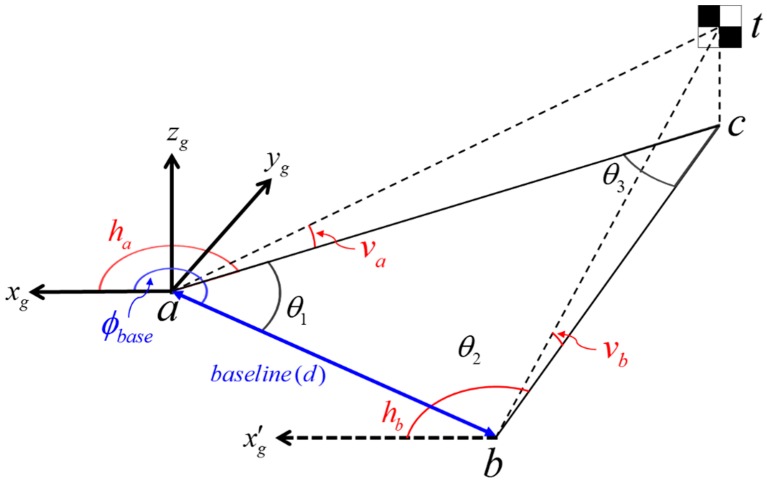
3D triangulation for target detection using a total station.

A prerequisite for calibration is the determination of the coordinates of the target centers using a high-accuracy independent measurement technique [12]. In the present research, both the horizontal and vertical target arrays were first surveyed with the total station TOPCON GPT-9000A (accuracy range: 0.3~1.5 *mgon* according to minimum reading (1 *mgon* equals to 3.24″)), then the target centers were calculated using the least squares adjustment. Though the total station offers the non-prism mode, its distance accuracy can be influenced by surface reflection and steep incidence angles to the target. Alternatively therefore, the target centers’ coordinates were obtained by manual reading of azimuth measurements. In Figure 11, points *a* and *b* indicate the locations of the two total station’s setups in the global frame. In the experiment, the position of *a* also was determined according to the origin of the global frame, and the position of *b* was measured by the total station at *a*.

In the adjustment, the two horizontal angle ($h_{a}$ and $h_{b}$) and two vertical angle ($v_{a}$ and $v_{b}$) measurements from stations a and b, along with the additional distance measurement (*d*) of the baseline (obtained by measuring tape to achieve high redundancy for adjustment), were adjusted simultaneously using the least squares method. The observation equations for the distance ($F_{d}$), horizontal ($F_{h}$) and vertical ($F_{v}$) azimuth measurements are given by:

(6) $$F_{d}=\sqrt{{\left(x_{t}-x_{i}\right)}^{2}+{\left(y_{t}-y_{i}\right)}^{2}}$$

(7) $$F_{h}=\tan^{-1}\left(\frac{x_{t}-x_{i}}{y_{t}-y_{i}}\right)$$

(8) $$F_{v}=\tan^{-1}\left(\frac{z_{t}-z_{i}}{\sqrt{{\left(x_{t}-x_{i}\right)}^{2}+{\left(y_{t}-y_{i}\right)}^{2}}}\right)$$

where $x_{i}$, $y_{i}$, $z_{i}$ are the fixed coordinates of the *i*th station (*a* or b), and $x_{t}$, $y_{t}$, $z_{t}$ are the coordinates of the target center to be adjusted. The Jacobian matrix (*J*), which contains the partial derivatives of the observation equations with respect to the target center coordinates set, is formed as:

(9) $$\left[\begin{array}{ccc} \frac{\partial F_{d}}{\partial x_{t}} & \frac{\partial F_{d}}{\partial y_{t}} & 0\\ \frac{\partial F_{h_{a}}}{\partial x_{t}} & \frac{\partial F_{h_{a}}}{\partial y_{t}} & 0\\ \frac{\partial F_{h_{b}}}{\partial x_{t}} & \frac{\partial F_{h_{b}}}{\partial y_{t}} & 0\\ \frac{\partial F_{v_{a}}}{\partial x_{t}} & \frac{\partial F_{v_{a}}}{\partial y_{t}} & \frac{\partial F_{v_{a}}}{\partial z_{t}}\\ \frac{\partial F_{v_{b}}}{\partial x_{t}} & \frac{\partial F_{v_{b}}}{\partial y_{t}} & \frac{\partial F_{v_{b}}}{\partial z_{t}} \end{array}\right]\left[\begin{array}{c} {\hat{\delta }}_{x_{t}}\\ {\hat{\delta }}_{y_{t}}\\ {\hat{\delta }}_{z_{t}} \end{array}\right]+\left[\begin{array}{c} u_{d}\\ u_{h_{a}}\\ u_{h_{b}}\\ u_{v_{a}}\\ u_{v_{b}} \end{array}\right]=\left[\begin{array}{c} {\hat{\upsilon }}_{d}\\ {\hat{\upsilon }}_{h_{a}}\\ {\hat{\upsilon }}_{h_{b}}\\ {\hat{\upsilon }}_{v_{a}}\\ {\hat{\upsilon }}_{v_{b}} \end{array}\right]$$

where $\hat{\delta }$ indicates the vector of the corrections to the approximate values for the target coordinates, u represents the calculated minus observed values, and $\hat{\upsilon }$ represents the estimated residuals. In the unweighted least squares adjustment, the solution of the adjustment model and its covariance matrix can be obtained as:

(10) $$\hat{\delta }={\left(J^{\text{T}}J\right)}^{-1}J^{\text{T}}u$$

(11) $$S^{2}=S_{0}^{2}{\left(J^{\text{T}}J\right)}^{-1}$$

where $S_{0}^{2}$ is the reference variance. The standard deviations are derived from the square root of the diagonal elements of the covariance matrix [30].

**Figure 12:**
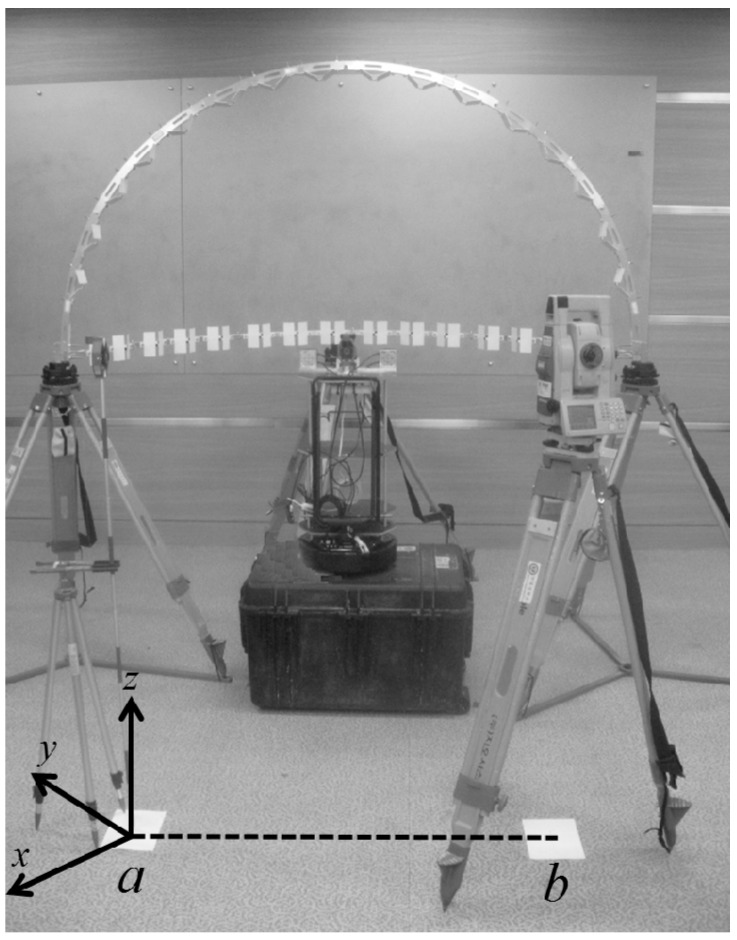
Surveying of calibration targets by a total station.

Figure 12 shows the total station surveying. Point *a* indicates the first total station setup as well as the origin of the global frame. Point *b* was setup to have redundant measurements for network adjustment: Its 2D position and orientation with respect to the origin *a* were determined according to the prism target. The dashed line *ab* indicates the baseline. In the experiment, some targets were not measured by the total station, due to steep incidence angles; thus, the number of targets (16 targets on each frame) was chosen in consideration of the viewing angle from each total station setup. Subsequently, the position of the scanning system in the global frame was measured using a prism target on the platform base, as shown in Figure 9b.

The adjusted positions and their estimated standard deviations are listed in Table 2 for the horizontal targets and in Table 3 for the vertical targets.

**Table 2:** Positions and standard deviations of the horizontal targets.

	Position (m)			Standard Deviation (mm)		
No.	*x*	*y*	*z*	*x*	*y*	*z*
1	0.348	1.432	1.188	0.102	0.367	0.116
2	0.472	1.541	1.189	0.069	0.236	0.076
3	0.630	1.634	1.187	0.070	0.218	0.074
4	0.781	1.688	1.188	0.099	0.279	0.097
5	0.939	1.715	1.189	0.067	0.167	0.060
6	1.125	1.715	1.190	0.091	0.202	0.074
7	1.283	1.688	1.194	0.193	0.379	0.141
8	1.434	1.635	1.197	0.161	0.284	0.108
9	1.596	1.543	1.197	0.068	0.106	0.042
10	1.722	1.445	1.195	0.154	0.215	0.088
11	1.829	1.326	1.195	0.180	0.227	0.096
12	1.930	1.168	1.194	0.133	0.149	0.066
13	1.992	1.020	1.193	0.115	0.118	0.054
14	2.030	0.866	1.193	0.246	0.230	0.108
15	2.037	0.680	1.192	0.226	0.192	0.094
16	2.015	0.516	1.191	0.220	0.172	0.087

**Table 3:** Positions and standard deviations of the vertical targets.

	Position (m)			Standard Deviation (mm)		
No.	*x*	*y*	*z*	*x*	*y*	*z*
1	0.234	1.226	1.444	0.041	0.150	0.051
2	0.284	1.201	1.602	0.096	0.322	0.166
3	0.367	1.155	1.759	0.129	0.377	0.269
4	0.461	1.104	1.877	0.133	0.333	0.290
5	0.571	1.042	1.976	0.045	0.095	0.096
6	0.716	0.965	2.066	0.032	0.058	0.066
7	0.849	0.891	2.117	0.088	0.142	0.176
8	0.988	0.816	2.143	0.162	0.241	0.313
9	1.152	0.726	2.143	0.252	0.354	0.469
10	1.291	0.650	2.116	0.329	0.443	0.580
11	1.423	0.577	2.065	0.449	0.569	0.726
12	1.568	0.498	1.976	0.564	0.645	0.780
13	1.679	0.437	1.877	0.575	0.590	0.663
14	1.773	0.386	1.759	0.489	0.448	0.446
15	1.857	0.340	1.603	0.643	0.524	0.411
16	1.906	0.311	1.446	0.165	0.124	0.069

In the tables, the target number 1 indicates the first target from the left on each aluminum frame in Figure 12. Overall, the standard deviations were calculated to be less than 0.4 mm for the horizontal targets and 0.8 mm for the vertical targets.

## 4. Experimental Results

**Figure 13:**
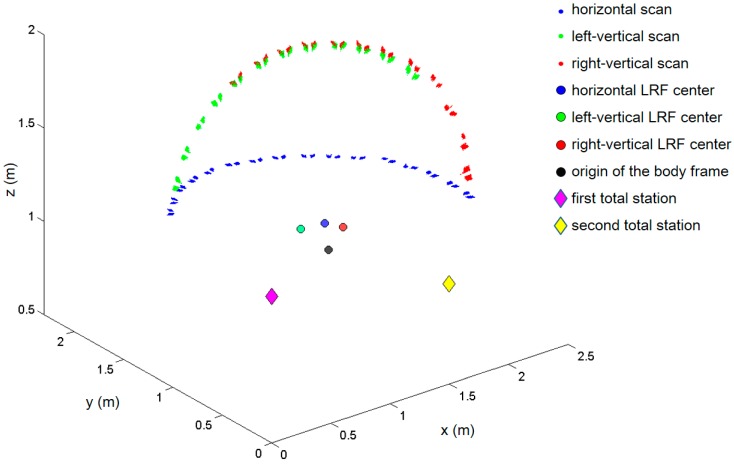
Point-cloud data acquisitions by the developed scanning system.

The calibration facility was designed for a self-assembled structure; thus it can be set up in any open place. Figure 13 shows the point-cloud data acquisitions (100 scans) by the developed scanning system. The diamond marks represent the two total station setups, where the blue mark indicates the reference station defining the origin of the global frame. The origin of the platform body frame is represented by the large black point, and the large green, blue, and red points indicate the locations of the left-vertical, horizontal, and right-vertical LRF sensors, respectively. The point-cloud groups also are displayed, the individual colors corresponding to each sensor. Whereas the horizontal sensor scanned all of the 16 horizontal targets, the two vertical sensors scanned only 12 targets each (red and green), due to the limited scanning range.

**Figure 14:**
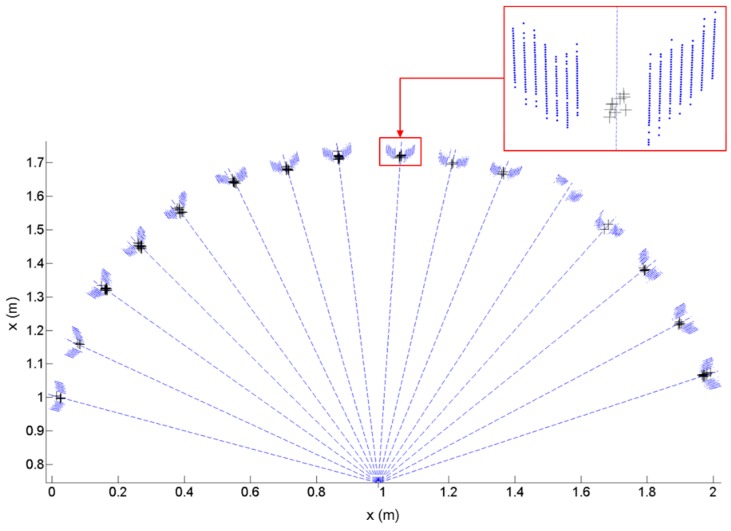
Estimation of the horizontal target centers by laser scanning.

Figure 14 shows the point clouds of the horizontal targets on the *x-y* plane (100 scans). Each target center is identified by dotted lines connected to the scanner origin. In order to take only the point returns from the target surfaces, the scanned points beyond the range of $\theta \;\pm \;2.5^{\circ }$ (θ is the direction angle from the scanner origin to each target center, as defined by the dashed line) were filtered out. Each target center was then calculated from the two intersecting scan lines on a target surface. Owing to the high standard deviations of the Hokuyo sensors (10 mm at 0.1 to 10 m range), some intersecting points were calculated far from their original target centers, particularly for the scans on the right side (possibly due to sensor imperfections), which later lead to significant errors in adjustment. This could be prevented by discarding the intersecting points beyond the range of $\theta \;\pm \;0.25^{\circ }$ (the hollowed part of the target in Figure 10). In Figure 14, the remaining intersecting points are indicated by the ‘+’ symbol.

As noted earlier, because it was assumed that the lever-arm parameters were known from the design drawing of the sensor stand, the bore-sight angles were the main concern of the multiple-scanner calibration. Once the design matrix was formed from the measurements by the total station and the scanning system, the calibrated parameters were computed using Equations (3)–(6). The residuals were then computed by substitution of the estimated values for the observed values. In practice, a large number of target observations (from 1000 scan lines) are collected to provide with high redundancy in the adjustment, and the parameter estimates are further improved. To initialize the iterative solution, the initial values for the bore-sight parameters were derived from the design drawing (Figure 3), and are listed in Table 4. The convergence criterion for all of the parameters was set to 10^−5^. In the present study, two calibrations, without and with the constraint, were conducted and compared by correlation analysis, because the correlations among estimated parameters are considered to be a good indicator of the adjustment quality [27].

**Figure 15:**
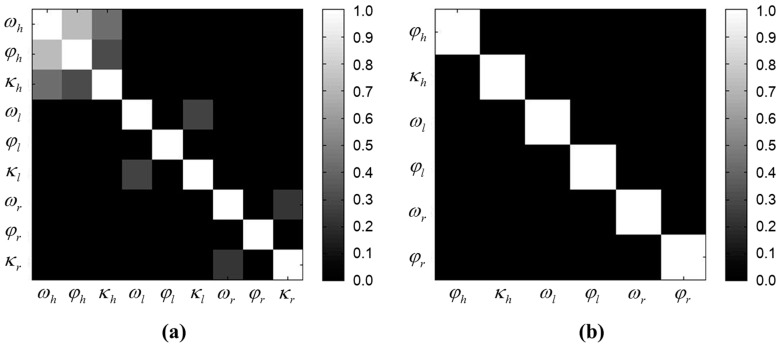
Correlation matrix of the calibrated parameters (**a**) without and (**b**) with the constraints.

Figure 15a shows the correlation matrix of calibration parameters without the constraint, where each color represents a different range of correlation coefficient magnitude: The bright color indicates high correlations, and the dark color, *vice versa*. The horizontal scanner’s parameters, particularly for $\omega_{h}$ and $\varphi_{h}$, were highly correlated (the correlation was about 0.723), which can incur failure in the adjustment. This high correlation can be explained by the weak network geometry of the horizontal targets; indeed, as shown in Table 2, the *z*-value variation was very low (less than 10 mm). However, this configuration was inevitable, because the *z*-values of the horizontal targets were leveled precisely for detection by the horizontal scanning. The two vertical scanners also showed correlations, albeit small, between $\omega_{l}$ and $\kappa_{l}$ (0.278), and between $\omega_{r}$ and $\kappa_{r}$ (0.219).

In order to achieve de-correlation between parameters, the constrained least squares approach was employed. Assuming the horizontal scanner to be set at a fixed flat level on the sensor stand, the calibration of parameter $\omega_{h}$ was excluded from the adjustment by enforcing the condition $\Delta \omega_{h}=0$. Likewise, the correlated parameters $\kappa_{l}$ and $\kappa_{r}$ in each vertical scanner were removed from the adjustment. Consequently, the correlation matrix (Figure 15b) showed sufficient de-correlation among the remaining six parameters.

The initial approximations and the calibrated parameters without and with the constraints are listed in Table 4. In the calibration without constraints, the adjustment was terminated by force after 10 iterations because it diverged. Compared with the initial approximations, the most noticeable change was found with $\omega_{h}$, which showed the great offset value of −144.902°. This was virtually impossible in consideration of the horizontal scanner’s position on the platform base (Figure 2 and Figure 6); this result therefore was considered an adjustment failure due to the high correlation between the horizontal sensor parameters. With the constraints, the calibration converged in four iterations, representing the largest changes in parameter $\omega_{l}$ (−1.018°) for the left and in $\omega_{r}$ (−1.017°) for the right vertical scanner.

**Table 4:** Calibration parameters (unit: °).

Calibration Parameters	Initial Approximation	Calibration without Constraints	Calibration with Constraints
$\omega_{h}$	0.000	−144.902	0.000
$\varphi_{h}$	0.000	−0.100	−0.148
$\kappa_{h}$	−90.000	−90.625	−90.274
$\omega_{l}$	0.000	−1.018	−1.018
$\varphi_{l}$	90.000	90.004	90.004
$\kappa_{l}$	0.000	−0.001	0.000
$\omega_{r}$	0.000	−1.194	−1.017
$\varphi_{r}$	90.000	89.969	89.965
$\kappa_{r}$	−180.000	−179.180	−180.000
**Convergence**	-	No	Yes

**Figure 16:**
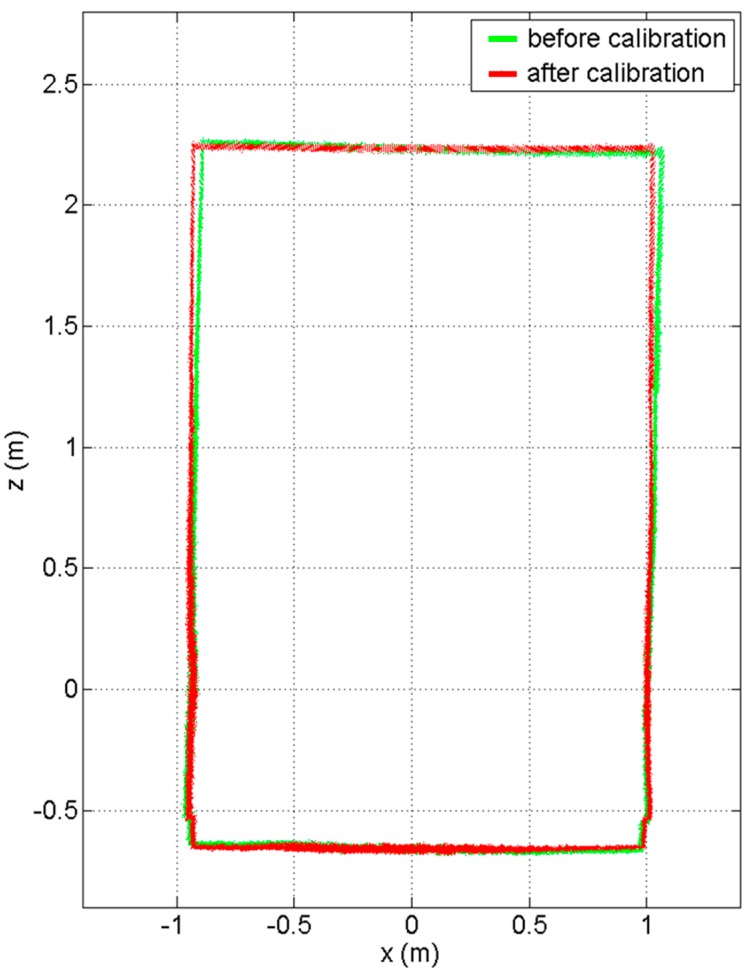
Cross-section view of the point-cloud data acquisition before (green) and after the constrained calibration (red).

Figure 16 shows a comparison of the mapped data (a cross-section view of the corridor with 30 scans) before and after the constrained calibration. The green points represent the scanned points with the initial approximations, and show that the profile was tilted to the right due to the rotation errors in $\omega_{l}$ and $\omega_{r}$. By contrast, the red points, which were acquired with the calibrated parameters resulting from the constrained adjustment, show that the tilt problem was resolved after calibration.

**Figure 17:**
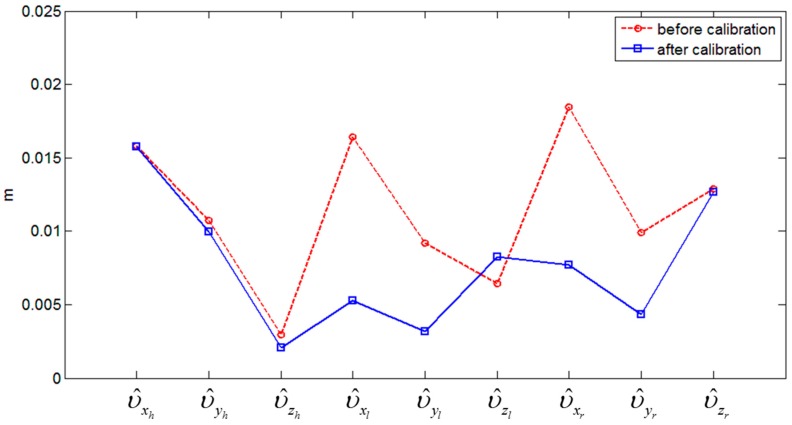
Estimated mean residuals before and after the constrained calibration.

Finally, Figure 17 shows the estimated mean residuals before and after the constrained calibration. The red line indicates the mean residuals estimated with the initial approximations, and the blue line indicates those estimated with the calibrated parameters resulting from the constrained adjustment. The considerable reductions achieved through the calibration procedure are clearly evident in the plotted results, particularly for the two vertical scanner parameters. Contrastingly, it was found that the residual ${\hat{\upsilon }}_{z_{l}}$, which was related to the constrained calibration parameter $\kappa_{r}$, was increased slightly, since the corrections were optimized for the other, non-constrained parameters in the adjustment.

## 5. Conclusions

A kinematic 3D laser scanning system was developed and calibrated using a specially designed double-deck calibration facility. The employed calibration approach stemmed from the bore-sight self-calibration approach used in photogrammetry. The double-deck calibration facility was designed for multi-LRF calibration, and its two-panel target allows for automated detection of the intersection of two scan lines, thus resulting in high redundancy and more rigorous calibration. Further, in order to achieve sufficient de-correlation between the parameters, the constrained least squares adjustment was applied. The experimental result demonstrated that the calibration had improved the point-cloud data acquisitions by kinematic scanning, in terms of both visual inspections and the measurement residuals after adjustment.

The proposed calibration facility and methodology are not limited to the present scanning system. For the purpose of 3D mapping, several studies have been found to use the same multi-LRF configuration: one mounted horizontally on the mobile system for navigation, and the other mounted vertically to map the surrounding environment [8,9]. The proposed calibration facility and methodology can be effectively applied to calibrate such configurations. Moreover, the calibration facility has been designed to adjust its vertical frame’s slope using two screws on each side, allowing the calibration of the LRF sensor inclined at various angles as well. The proposed constrained least squares calibration, under the predefined assumptions, can be used simply to handle problems in estimation such as highly correlated parameters.

Nevertheless, the proposed constraint approach is not the best way to reduce strong correlations among parameters, because it cannot adjust the constrained values. Moreover, in the present study, calibration for the intrinsic and lever-arm parameters was not considered, because such augmentation can result in additional correlation among those parameters. In order to achieve sufficient de-correlation then, future work should include the design of a new calibration facility ensuring a better network geometry in a 3D context, as well as the testing of various types of targets. Additionally, the influences of target characteristics such as surface brightness, color and material on the calibration also need to be taken into consideration.
